# Cardiovascular Outcomes of PCSK9 Inhibitors: With Special Emphasis on Its Effect beyond LDL-Cholesterol Lowering

**DOI:** 10.1155/2018/3179201

**Published:** 2018-03-25

**Authors:** Dhrubajyoti Bandyopadhyay, Kumar Ashish, Adrija Hajra, Arshna Qureshi, Raktim K. Ghosh

**Affiliations:** ^1^Internal Medicine, Mount Sinai St Luke's Roosevelt Hospital Center, New York, NY, USA; ^2^University of Texas MD Anderson Cancer Center, Houston, TX, USA; ^3^IPGMER, Kolkata, India; ^4^Department of Medicine, Lady Hardinge Medical College, New Delhi, India; ^5^Metro Health, Case Western Reserve University, Cleveland, OH, USA

## Abstract

PCSK9 inhibitors, monoclonal antibodies, are novel antihypercholesterolemic drugs. FDA first approved them in July 2015. PCSK9 protein (692-amino acids) was discovered in 2003. It plays a major role in LDL receptor degradation and is a prominent modulator in low-density lipoprotein cholesterol (LDL-C) metabolism. PCSK9 inhibitors are monoclonal antibodies that target PCSK9 protein in liver and inhibiting this protein leads to drastically lowering harmful LDL-C level in the bloodstream. Despite widespread use of the statin, not all the high-risk patients were able to achieve targeted level of LDL-C. Using PCSK9 inhibitors could lead to a substantial decrement in LDL-C plasma level ranging from 50% to 70%, either as a monotherapy or on top of statins. A large number of trials have shown robust reduction of LDL-C plasma level with the use of PCSK9 inhibitors as a monotherapy or in combination with statins in familial and nonfamilial forms of hypercholesterolemia. Moreover, PCSK9 inhibitors do not appear to increase the risk of hepatic and muscle-related side effects. PCSK9 inhibitors proved to be a highly potent and promising antihypercholesterolemic drug by decreasing LDL-R lysosomal degradation by PCSK9 protein. Statin drugs are known to have some pleiotropic effects. In this article, we are also focusing on the effects of PCSK9 inhibitor beyond LDL-C reduction like endothelial inflammation, atherosclerosis, its safety in patients with diabetes, obesity, and chronic kidney disease, and its influence on neurocognition and stroke.

## 1. Introduction

Heart disease is the leading cause of death in the US (23.7% of total deaths in 2011) [1]. Approximately one out of three Americans died of heart disease and stroke [2]. People with high cholesterol level are twice more likely to be suffering from heart disease than normal adults. 73.7 million or 31.7% of US adults are found to have high LDL-C. Currently, near about half of the adults (48.1%) with elevated LDL-C is getting treatment. Less than one-third (29.5%) of the population with high LDL-C is under control [1]. Familial hypercholesterolemia (FH) which is due to the mutation of specific LDL receptor gene has been found in 1 in 299 population in the US [3]. In the case of homozygous FH, the cholesterol level can be elevated even up to 1000 mg/dl (with LDL-C > 600 mg/dL) and in heterozygous FH this level may reach up to 350–550 mg/dl (with LDL-C = 200–400 mg/dL). Patients with untreated FH are prone to develop widespread atherosclerosis from their early life. Most of the untreated homozygous FH patients usually develop heart attack in their late teens and about half of the heterozygous FH suffer from heart disease at around 45 years for men and 55 to 60 years for females [4, 5]. According to 2013 AHA/ACC guidelines individuals with LDL-C level more than 190 mg/dl require high-intensity statin therapy to achieve 50% reduction. It is noteworthy that maximally tolerated dose of statin even with the combination of other nonstatin cholesterol-lowering medications is not sufficient to attain this goal, particularly in the case of FH [6]. In a study only 21% of patients achieved the target LDL-C level with the use of statin as a single agent [7] and a data from the UK showed among patients using combination therapy (statin and ezetimibe) only 44% patients achieved the target LDL-C level [8].

## 2. Existing Lipid-Lowering Agents

The primary lipid-lowering agents include the statin, ezetimibe, bile acid sequestrants, nicotinic acid, and fibrates. Among them, statin, ezetimibe, and bile acid sequestrants are mainly used to lower LDL-C level. Statin acts by inhibition of HMG-CoA reductase, thereby increasing LDL receptor activity. Ezetimibe inhibits cholesterol absorption by inhibiting Niemann-Pick C1-like 1 protein. Nicotinic acid and fibrates are popularly known for their triglyceride reducing property [5]. Statin is widely used to lower LDL-C and thus for primary and secondary prevention of cardiovascular disease. But this effect does not come without any side effect. Hepatic dysfunction (seen in 0.5 to 3.0% of patients) [9], myopathy (approximately 0.1% of patients develop myopathy) [10], myositis and rhabdomyolysis (near about 5% patients develop statin-associated muscle symptoms) [11], proteinuria, acute kidney injury [12], cognitive changes [13], induction of diabetes mellitus, rare cases of neuropathy [14], and drug-induced lupus have been reported [9]. In the US, the statin is considered as category X in pregnancy [9]. Overall statin intolerance is seen approximately in 10–15% of patients in clinical practice [15]. Statin is not sufficiently useful in patients with very high plasma levels of LDL-C including FH patients and patients with elevated plasma levels of lipoprotein(a) even with combination with ezetimibe. Most of the cases are due to statin intolerance or their LDL-C levels are too high to control with statin-dependent therapy. So there is a pressing need to think beyond statin in such patients.

## 3. Newer Hypolipidemic Drugs Either Approved Recently Or in Late Stage Development

Recently several new classes of lipid-lowering drugs have been evolved.

Lomitapide, approved by the FDA in December 2012, is an inhibitor of microsomal triglyceride transfer protein (MTP). It is used orally and indicated mainly in homozygous FH or severe heterozygous FH [16].

Mipomersen (inhibitor of apolipoprotein B-100), an antisense oligonucleotide complementary to the coding region of human apo-B mRNA, was approved by the FDA in January 2013. It is used subcutaneously in FH patients mainly [16].

Inhibitors of cholesteryl-ester transfer protein (CETP) causes increase HDL and decrease LDL-C by 40–45% [17]. According to REVEAL trial on anacetrapib, use of CETP inhibitors in the patient with atherosclerotic vascular disease along with intensive statin regime resulted in lower incidence of major coronary events compared to the placebo arm [18]. Bempedoic acid (ETC-1002), a novel small molecule, is known to affect carbohydrate and lipid metabolism and reduce the LDL-C level near about 27% [19].

Proprotein convertase subtilisin/kexin type-9 (PCSK9) inhibitors are monoclonal antibodies which bind to the PCSK9 protein and regulate LDL-C level in blood. Alirocumab (Praluent, marketed by Sanofi-Aventis) and evolocumab (Repatha, sold by Amgen Inc.) have been approved by US-FDA in July and August 2015, respectively. Bococizumab (RN316, Pfizer) was undergoing cardiovascular safety trial, and, after showing inadequate results, the trials have been stopped, and Pfizer also discontinued its production [20].

## 4. PCSK9 and Cholesterol Pathway in the Body

The discovery of proprotein convertase subtilisin kexin 9 (PCSK9) by Abifadel et al. in the year 2003 has revolutionized the management of FH and subject not responding to statins regime [21]. PCSK9 gene is located on chromosome 1p32.3 [22]. PCSK9, a serine protease, is mainly produced by the liver, intestine, and kidney. PCSK9 is synthesized as 692-amino acid protein (73-kDa zymogen). After intramolecular autocatalytic processing in the endoplasmic reticulum (ER) 73-kDa zymogen gives rise to a 14-kDa prodomain and a 63 kDa mature PCSK9 [23]. Under normal circumstances, the binding of LDL to its receptor (LDL-R) is followed by the endocytosis of the complex by endosomes. At the plasma membrane, PCSK9 interacts with the LDL-R, but the neutral pH negatively modulates this interaction. On the contrary, the acidic pH of the endosome increases the affinity of the two by manifolds. Consequently, the positively charged C-terminal domain of PCSK9 binds to the negatively charged ligand-binding domain of the LDL-R [24–26] and thereby locks the LDL-R in an open conformation. The failure to attain a closed conformation in the endosome prevents normal recycling of the LDL-R to the plasma membrane. The LDL-R is then routed to lysosomes for degradation [24, 27]. As a consequence of decreased recycling, LDL-R at the cell surface is attenuated, and so does the LDL-C clearance. This normal physiology is magnified by gain-of-function mutations of PCSK9 leading to elevated LDL-C level and cardiovascular disease (CVD). Loss-of-function mutations of the PCSK9 result in increased surface LDL-R and improved LDL-C clearance [28].

The inverse relation between PCSK9 activity levels and LDL-R suggests that PCSK9 inhibition could have a synergistic effect with statins on LDL-C.

PCSK9 gene mutation is implicated in approximately 1-2% of patients with FH. PCSK9 gene mutation is the third commonest cause of FH, after LDL receptor or apolipoprotein B (ApoB) genes mutation [29]. The loss-of-function mutation of PCSK9 gene exhibited mitigation of CVD risk by 88% in the black population [30]. This observation fueled the concept that PCSK9 inhibitor might be beneficial in cases of FH and CVD.

## 5. Why PCSK9 Inhibitors Have Great Potential?

Patient population with FH is relatively small. The prevalence of heterozygous FH and homozygous FH due to loss of function of various gene are estimated as 1 in 500 population and 1 in 1 million, respectively [5].

However, some patients who are intolerant of statin treatment as high as 3 million (or up to 15% of patients taking statins) [31]. Till now vitamin and minerals like coenzyme-Q10 supplementation do not appear to prevent statin-induced muscular problems [32].

The number of the patients on statins but not achieving the target LDL-C levels would be even higher (only about one-third patient will achieve target LDL-C < 70 mg/dl even with high dose statin) [33]. In statin-treated patients there is upregulation of PCSK9 which attenuates the efficacy of statin. This observation led to the development of PCSK9 inhibitor [34]. On the other hand, PCSK9 inhibitor lowers LDL level in a dose-dependent manner. It reduces LDL-C by 70 percent and 60 percent in statin naïve patients and patients currently on statin therapy, respectively [35]. This reduction of LDL-C has been proven to have significant benefits in clinical studies irrespective of baseline cardiovascular risks.

FOURIER trial on evolocumab, a randomized, double-blind, placebo-controlled trial enrolling 27,564 participants with history of atherosclerotic disease, has shown a substantial reduction of all cause of mortality, cardiovascular mortality, and myocardial infarction with use of evolocumab on a background of statin therapy [36]. Heightened risk of major adverse cardiac (MACE) and limb events (MALE) typically present in patient with symptomatic peripheral arterial disease. As per the results of subanalysis study of FOURIER trial, evolocumab also mitigates the risk of MALE and the relation between achieved LDL-C and lower limb events are directly proportional. Thus, reduction of LDL-C to extremely low level should be considered in a subjects with PAD, regardless of history of MI or stroke, to diminish the chance of MACE and MALE [37].

## 6. A Brief Description of PCSK9 Inhibitors

*Alirocumab* (approximate molecular weight of 146 kDa), a human monoclonal antibody (IgG1), consists of two disulfide-linked heavy chains which are disulfide-linked to a light chain [38, 39]. It is used at a dose of 75–150 mg subcutaneously once every two weeks. The onset of action is 4–6 hours, and elimination half-life is usually 17–20 days. It undergoes proteolysis in many tissues to form polypeptides and amino acids [40].

*Evolocumab* (approximate molecular weight of 141.8 kDa), a human monoclonal antibody (IgG2) lambda with gamma 2 heavy chain linked by a disulfide bond to lambda light chain. It is administered subcutaneously at a dose of 140 mg every two weeks or 420 mg once monthly. The onset of action is within 4 hours and half-life of elimination is 11–17 days. It is metabolized by nonsaturable proteolysis [41, 42].

*Bococizumab* (approximate molecular weight of 145.1 kDa) is a humanized monoclonal antibody IgG2-Kappa with gamma 2 heavy chain linked by disulfide bond with kappa light chain. It is administered at a dose of 150 mg every two weeks or 300 mg once monthly by subcutaneous injection [43].

## 7. Brief Preclinical Studies

In mice lack of PCSK9 is protective against atherosclerosis and overexpression of it causes increased accumulation of cholesteryl-esters in aorta leading to accelerated atherosclerosis [44]. Alirocumab reduced atherosclerosis lesion size, monocyte and T-cell recruitment, smooth muscle cells proliferation, collagen, and macrophage content, thus improving plaque morphology in mice [45]. Infection and inflammation play a key role in the expression of PCSK9 in mice model. Clearance of LPS requires LDL or HDL binding as transport protein, and PCSK9 inhibition leads to increase expression of LDL-R causing more clearance of LDL along with LPS. This finding highlights the anti-inflammatory action of PCSK9 inhibitors [46]. Preclinical studies by Walley et al. and Dwivedi et al. also supported the anti-inflammatory PCSK9 inhibitors in mice animal model [47, 48]. Alirocumab administration in mice has shown reduced circulating neutrophil, monocytes, and decreased expression of endothelial ICAM-1. Thus it attenuates monocytes attachment to vascular endothelium and dampens vascular inflammation. Food intake, body weight, and weight of the liver were unaltered with alirocumab therapy in mice [45]. Reversible liver parenchymal hypertrophy and nonsignificant adrenal cortex hypertrophy have been reported in animals. There are no increased risks of hepatitis-C virus infection, immunosuppression, neurocognitive dysfunction, type 2 diabetes mellitus, and no increased bile acid concentration in intestine, thus possessing no significant increased risk of the intestinal tumor in the animal model [49]. In rats, bococizumab administration demonstrates no adverse effects on embryo-fetal development even in dose greater than usual clinical dosage [50].

## 8. Clinical Development of PCSK9 Inhibitors

### 8.1. Alirocumab

Alirocumab is a novel PCSK9 inhibitor. Many studies have been done and still going on to find out its efficacy and safety profile which have been summarized in Table 1. Its LDL lowering effect is also independent of the site of injection [51].

Also, a pooled data from 3 double-blind, randomized, placebo-controlled, phase 2 studies showed alirocumab to reduce LDL-apo® and Lp(a) significantly from baseline in comparison to placebo [52]. As we know that lipoprotein is an independent risk factor for CAD, this finding holds promise [53].

### 8.2. Evolocumab

Several clinical studies have established the efficacy and safety of evolocumab [Table 2]. GAUSS-3 Randomized Clinical Trial also proved its tolerability in patients with muscle-related statin intolerance [54].

### 8.3. Bococizumab

Despite the fact that the initial studies showed promising result [Table 3], recently SPIRE trials showed attenuation of the effect of bococizumab in 15–20% of patients due to the formation of antibody against its murine component. This led to the interruption of further development of this drug [55].

## 9. The Role of PCSK9 beyond LDL-C Lowering

PCSK9 inhibitors after being established as a valid option, now its effects on inflammation, endothelial function, atherosclerosis, diabetes, and obesity are now actively investigated. 

*(i) The PCSK9 Level in CKD Patients and HD Patients*. In a study, it was shown that serum PCSK9 level was decreased in chronic kidney disease patients who were on hemodialysis (CLD-HD) and PCSK9 had a positive correlation with LDL-C level. This signifies that PCSK9 plays a major role in regulating LDL-C even in CKD-HD patients. PCSK9 also is involved in the metabolism of triglyceride-rich lipoproteins in CKD-HD patients [56]. PCSK9 level tends to rise in patients with nephrotic syndrome, and it has a positive correlation with proteinuria. The PCSK9 level is also higher in patients on peritoneal dialysis in comparison to hemodialysis or renal transplant patients [57]. 

*(ii) PCSK9 and Lipoprotein A.* Lp (a) is a widely accepted cardiovascular risk factor, except for regular extracorporeal lipoprotein apheresis which is the only available modality to reduce Lp (a). The possible mechanism of Lp (a) reduction with use of PCSK9 inhibitors is because of the enormous expression of LDL-R due to PCSK9 inhibition, which unmasks clearance mechanism of Lp (a) by abundant hepatic LDL-R [58]. Additionally, Canuel et al. have reported that PCSK9 degrades LDL related protein-1, which catabolizes Lp(a). Thus, the inhibition of PCSK9 increases the catabolism of Lp(a) [59].

Alirocumab also reduces non-HDL cholesterol and fasting triglycerides significantly. Alirocumab also increases the HDL level and apolipoprotein A-1. Though the possible mechanism which increases HDL-C is not entirely clear, one likely hypothesis is that reduction of LDL-C causes reduction in cholesteryl-ester transfer protein activity, as less LDL-C is available to transfer out cholesterol from HDL particles. Eventually, it causes a relatively high level of HDL-C [60]. 

*(iii) PCSK9 Inhibitors on Inflammation and Atherosclerosis*. Atherosclerosis is a chronic inflammatory process within the arterial wall. Proinflammatory effect of PCSK9 has been shown in different experimental models. This effect is supposed to be responsible for promoting atherosclerosis independent of LDL-C level. Vascular smooth muscle cells (VSMC), oxidized LDL-C, have shown a high level of PCSK9 expression [61].

LOX-1, a receptor for oxidized LDL in VSMC, is upregulated in inflammation. It has been reported that PCSK9 stimulates transcription of LOX-1 and LOX-1, in turn, and stimulates PCSK9 expression, which facilitates atherogenesis [62]. The interaction between PCSK9 and LDL-R favors the entry of inflammatory monocytes into the arterial wall and thus promotes atherosclerosis [63]. Though it had been shown in a study that there is no effect of PCSK9 inhibitors on hs-CRP concentration level [64], the relationship of PCSK9 with systemic inflammation cannot be denied. Walley et al. revealed that loss of PCSK9 function in both mice model and human enhances pathogen lipid clearance with the help of LDL receptor and regulates inflammatory response in septic shock with better survival [48].

ATHEROREMO-IVUS study conducted by Cheng et al. revealed a linear correlation between PCSK9 level and the amount of necrotic tissue in atherosclerotic plaque [65]. Lowering of LDL-C by PCSK9 inhibitors reduces inflammation, endothelial apoptosis, and the concentration of oxidized LDL-C within the plaque. This alters the composition of the plaque more favorably. GLAGOV Randomized Clinical Trial (*n* = 968), which was concluded last year, revealed that addition of evolocumab to statin-treated patient causes decrease in percent atheroma plaque volume assessed by sequential intravascular ultrasound after 76 weeks of therapy [66]. Macrophage recycles the cell membrane lipid from the dead cells including RBC. When recycling capacity of macrophages exceeds, those cell membrane lipids accumulate as atheroma. Macrophage fat catabolism capacity is associated with underlying atherosclerosis, and this could be quantified by accumulation acyl-carnitine intermediates in ECF which is the direct parameter of the adequacy of beta-oxidation to recycle membrane fatty acid. A study conducted by Blair et al. revealed that minimizing macrophage fat overload by reducing fat metabolism rate is favorable, which could be achieved by using statin and the newer PCSK9 inhibitors [67]. 

*(iv) PCSK9 in Diabetic Patients*. Diabetes is the major well-established risk factor for cardiovascular diseases. Diabetes increases the risk for atherosclerosis due to endothelial inflammation. Inflammation in blood vessels is one of the primary drivers for atherosclerosis and diabetes makes it much worse. A study conducted by Sattar et al. revealed that PCSK9 inhibitor markedly decreases atherogenic lipoproteins in diabetic patients and results are similar as seen in nondiabetic patients [68]. They did not alter the normal glucose homeostasis. Arsenault and colleagues have put the light on the fact that serum PCSK9 protein level is higher in insulin-resistant subjects, which also supports its temporal association of hyperlipidemia in diabetic patients [69]. The results from FOURIER trial demonstrated no significant difference in new-onset diabetes and neurocognitive events between evolocumab and placebo arm [36]. They reported similar levels of HbA1c and FPG between both the groups in patients with diabetes, prediabetes, and normoglycemia [70]. 

*(v) PCSK9 and Its Relation with Vitamin E, Cortisol, Adrenocorticotropic Hormone, and Gonadal Hormones.* Vitamin E is one of the important antioxidants which prevent oxidative damage of long-chain PUFA and cell membrane disruption. The function of vitamin E transport and steroidogenesis are intricately related to LDL-C metabolism. In a recent study, 901 patients with LDL-C ≥ 2.0 mmol/L were randomly assigned to monthly subcutaneous evolocumab for 52 weeks and placebo. In evolocumab-treated substudy group, the level of vitamin E in LDL-C was decreased substantially, and vitamin E level in HDL was increased significantly. Cortisol level was elevated in evolocumab-treated patients, but there were no changes in ACTH, cortisol : ACTH ratio, and gonadal hormone levels. So from this above data, we can conclude that in spite of lowering LDL-C level, evolocumab does not reduce the cholesterol normalized level of vitamin E, ACTH, and gonadal hormones [71]. 

*(vi) PCSK9 in Ischemic Stroke.* PCSK9 and LDL receptor are involved in mouse brain development. After 24 to 72 hrs. of reperfusion in ischemic stroke, PCSK9 is upregulated in the dentate gyrus of the mouse model but without affecting de novo neurogenesis. PCSK9 degrades the LDL receptor in the brain (telencephalon and cerebellum) both during development and ischemia/reperfusion. PCSK9 is expressed only in the olfactory peduncle in adult mice, but it does not degrade LDL receptor. In adult mice, blood-brain barrier is impermeable to PCSK9 which explains the absence of LDL receptor lowering effect of PCSK9 in the brain of adult mice. The effect of LDL receptor in the brain is still not clear. LDL receptor-negative mice show impaired learning and memory. Though our understanding of the effects of LDL receptor excess in the brain is still evolving, ablation of the PCSK9 gene in mice did not reveal any significant effect of decreased PCSK9 level on brain recovery after an ischemic stroke. So we can hope that PCSK9 inhibition by monoclonal antibody should not hamper brain recovery after an ischemic insult [72]. A meta-analysis involving 11 studies showed there is no increased incidence of stroke with its use [73]. 

*(vii) Effect of PCSK9 beyond Liver.* PCSK9 effect beyond LDL receptor degradation in the liver is largely unknown. PCSK9 is expressed by extrahepatic tissues such as intestine, pancreas, kidney, smooth vascular cells, endothelial cells, goblet cells, and brain. In insulin-resistant diabetic patient, PCSK9 level is significantly low in duodenum compared to insulin sensitive obese patient undergoing bariatric surgery. But it remains to conclude whether insulin induces expression of PCSK9 in the intestine as it does in the liver. PCSK9-deficient mice had sevenfold increased LDL receptor expression in intestine. PCSK9-deficient mice showed reduced postprandial hypertriglyceridemia due to reduced level of ApoB. A route of cholesterol excretion that is upregulated in the PCSK9-deficient mice leads to fecal cholesterol excretion. It is also involved in nephrogenesis and binds with amiloride-sensitive epithelial sodium channel (ENaC) and mediates their degradation by proteasome pathways. PCSK9 downregulates the LDL receptor expression on the surface of the isolated human pancreatic beta cell and in PCSK9 (−/−) mice; there were increased cell surface LDL receptors. But there is an inconsistency about the deleterious effect of LDL-C accumulation in beta cells insulin secretion of the PCSK9 (−/−) mice model. This discrepancy can be explained by the PCSK9 inhibitor-mediated reduction of LDL-C level which counterbalances the deleterious effect of LDL-C accumulation inside the beta cells [74]. 

*(viii) PCSK9 and Neurocognitive Effect.* In 2012, FDA issued a warning for all statin drugs: “ill-defined memory loss or impairment” [75]. The PCSK9 inhibitor is one of the most potent and promising new therapies to lower LDL-C level nowadays. So it is imperative to discuss any plausible role of it in causing neurocognitive impairment. LDL-R also is expressed in the brain and helps in clearing apolipoprotein-E, which is responsible for the formation of amyloid-*β* which accumulates in the brain of Alzheimer's disease patient. PCSK9 gene deleted mice have shown reduced apolipoprotein-E and amyloid-*β* formation. PCSK9-inhibitors cannot cross blood-brain barrier in human. Long-term LDL-C lowering by PCSK9 improves arterial health which in turn protects against the development of dementia. Though there are some studies which signal to unfavorable effects on neurocognition, recently Robinson et al. showed no increased neurocognitive risk in a pooled analysis of 14 trials on PCSK9 inhibitors even after attaining an extremely low LDL-C level [76]. Loss of function of PCSK9 is not associated with any symptoms of mental retardation as well.

Currently, few trials are ongoing to search any adverse neurocognitive effect of PCSK9 inhibitors, and we have to wait until the end of 2017-2018 for a final opinion regarding this aspect. 

*(ix) Available Safety Data on PCSK9 Inhibitors.* The most common adverse events occurring in alirocumab treated patients were gastrointestinal disorders, infections and infestations, musculoskeletal disorders, and skin and subcutaneous tissue disorders [77]. One patient with the history of atrial fibrillation and chronic obstructive pulmonary disorder had a pulmonary embolism after alirocumab therapy in ODYSSEY MONO study [78].

Nasopharyngitis, upper respiratory tract infection, influenza, and back pain are commonly found adverse effects regarding the use of evolocumab [79]. The increment of creatine kinase levels to more than five times the ULN occurred in 1.2% of patients in the evolocumab group in DESCARTES Trial [80]. Acute pancreatitis has been reported in MENDEL 2 trial as well [81].

Upper respiratory tract infection, nasopharyngitis, diarrhea, urinary tract infection, arthralgia, bronchitis, injection site erythema, gastroesophageal reflux disease, and cough are commonly found adverse effects after bococizumab use [82].

Interestingly, PCSK9 inhibitors do not lead to an increased rate of new-onset diabetes [83]. Though high blood glucose during the treatment period and baseline HbA1c of more than 6.5% have been found in alirocumab treated patients, there was no pattern in changes in either blood glucose or HbA1c from screening to week 24 [84]. Evolocumab treatment did not show any adverse effect on glycemic measures in a 52-week placebo-controlled trial [80].

A meta-analysis of 25 randomized controlled trials has shown no significant difference regarding the occurrence of adverse events between PCSK9 inhibitor group and placebo group (or ezetimibe group) [85].

Cholesterol is an important component of myelin protein. So, lipid-lowering therapies may play a role to hamper the neural structure and function. Along with that reduced serum cholesterol may also enhance the blood-brain barrier permeability. It may result in increased exposure of the central nervous system to the toxins in the blood. So there is a concern whether PCSK9 inhibitors can cause cognitive dysfunction by lowering cholesterol level [75]. A meta-analysis has shown a significant increase in neurocognitive events with PCSK9 inhibitor therapy in comparison with placebo therapy. But the analysis has some limitations due to lack of uniform data, heterogeneity of the studies, and lack of uniform definitions of the cardiovascular events [86]. On the other hand, LDL receptor causes clearance of apolipoprotein-E, a protein responsible for Alzheimer's disease. So PCSK9 inhibitors may have the protective role for this disease. Long-term PCSK9 inhibitor therapy can prevent vascular dementia by improving arterial health as well. So there may be a positive effect of PCSK9 inhibitors on neurocognitive functions. Cognitive side effects have been found as an uncommon finding in the OSLER study. Less than 1% patients showed amnesia and less than 1% patients showed mental impairment, but no precise data is available till now [75]. Apart from neurocognitive impairment, increased incidences of hemorrhagic stroke, hormonal insufficiency, and hemolytic anemia have been found to be associated with the very low LDL-C level [79]. There was only statistically insignificant increased risk of cataract in a recent analysis [76].

Pooled analysis from 10 ODYSSEY Trials established no increase adverse events in patients on alirocumab therapy [87].

Naturally more time-tested trials are required for conclusive data. To summarize, published evidence from the trials suggests that PCSK9 inhibitors are well tolerated and with good safety profile [84].

## 10. Conclusion and Future Direction

The discovery of PCSK9 proteins has changed the dynamics of lipid control in hypercholesterolemic patients. PCSK9 inhibitors pave the path of achieving an extremely low plasma level of LDL-C and have shown to reduce lifetime risk for CVD events. They are also involved in decreasing endothelial inflammation which is the key factor for atherosclerosis. Very aggressive lowering of LDL-C by PCSK9 inhibitors leads to plaque stabilization and regression. Large phase II and III trials for these monoclonal Abs have shown its safety, efficacy, and effectiveness in patients who are at risk for cardiovascular diseases due to dyslipidemia. Many trials have revealed that PCSK9 inhibitors have reduced all causes of mortality including cardiovascular mortality with less adverse effects like myopathy and hepatotoxicity. Though SPIRE trials were terminated early for the concern of immunogenicity, the immunogenicity of evolocumab is extremely low and neutralizing anti-drug antibody was seen only in 1.3% patients on alirocumab. Newer drug Inclisiran, a PCSK9-specific small interfering RNA, is also being studied. ODYSSEY OUTCOME study and other trials are also in progress to evaluate the whole spectrum of PCSK9 inhibitors, and the results are scheduled to come by the end of 2017 to 2018. It is to be evaluated by subsequent trials whether these pleiotropic effects would confer substantial morbidity and mortality benefits.

## Figures and Tables

**Table 1:** Trials on alirocumab. A-mAb: alirocumab; ATV: atorvastatin; EZE: ezetimibe; RSV: rosuvastatin; ASCVD: atherosclerotic cardiovascular disease; CHD: coronary heart disease; heFH: heterozygous familial hypercholesterolemia.

S. No	Trial [reference]	Participants	Comparison	LDL-C reduction	Comments
(1)	ODYSSEY MONO Completion date: July 2013 [50].	Patients with hypercholesterolemia	A-mAb versus EZE	47.2% versus 15.6%	-

(2)	ODYSSEY COMBO I Completion date: April 2014 [46].	Hypercholesterolemia + CHD or CHD equivalents, on treatment with maximal tolerated statin dose	A-mAb versus Placebo	48.2% versus 2.3%	-

(3)	ODYSSEY OPTIONS I Completion date: May 2014 [44].	Hyperlipidemia + risk of ASCVD, on baseline treatment with ATV	ATV + A-mAb versus ATV + EZE versus ATV (double dose) versus RSV	44.1% versus 20.5% versus 5.0% versus 21.4 %	-

(4)	ODYSSEY OPTIONS II Completion date: May 2014 [45].	Hyperlipidemia + risk of ASCVD, on baseline treatment with RSV	A-mAb versus EZE versus RSV	50.6% versus 14.4% versus 16.3%	-

(5)	ODYSSEY LONG TERM TRIAL Completion date: November 2014 [47].	Hypercholesterolemia + risk of ASCVD, on treatment with maximally tolerated statin dose	A-mAb versus placebo	61% versus 0.8%	-

(6)	ODYSSEY FH I Completion date: December 2014 [49]	Familial heterozygous hypercholesterolemia on maximally tolerated statin dose	A-mAb versus placebo	57.9% reduction in A-Mab group	-

(7)	ODYSSEY FH II Completion date: January 2015 [49]	Familial heterozygous hypercholesterolemia on maximally tolerated statin dose	A-mAb versus placebo	51.4% reduction in A-Mab group	-

(8)	ODYSSEY COMBO II Completion date: July 2015 [48].	Hypercholesterolemia + risk of ASCVD, on treatment with maximally tolerated statin dose	A-mAb versus EZE	50.6% versus 20.7%	-

(9)	ODYSSEY HIGH FH Completion date: 2016 Sep	Patients having heFH and LDL-C ≥ 160 mg/dl even after maximum tolerated dose of statin	A-mAb versus Placebo	45.7% versus 6.6%	

(9)	Phase 2 pooled analysis [51]	Primary hypercholesterolemia on lipid lowering therapy	A-mAb versus placebo	68.4% versus 10.5%	-

(10)	Randomized controlled trial [54]	Hypercholesterolemia on treatment with ATV	A-mAb versus placebo	40% to 70% versus 5%	-

(11)	Pooled analysis of 14 randomized controlled trials	_	A-mAb versus control (placebo or EZE)	LDL-C reduced to as low as 15 mg/dl in A-mAb group	Rates of adverse events in those achieving LDL-C < 25 mg/dl (72.7%) and <15 mg/dl (71.7%) were similar to those who did not (76.7%)

**Table 2:** Studies on evolocumab. E-mAb: evolocumab; EZE: ezetimibe; ATV: atorvastatin.

S. No.	Trial	Participants	Comparison	LDL-C reduction	Adverse events (AE)
(1)	MENDEL-2 Completion date: October 2013	Hypercholesterolemia	E-mAb versus Placebo versus EZE	Reduction in E-mab group; 55–57% more than placebo & 38–40% more than EZE	44% versus 44% versus 46%

(2)	DESCARATES Completion date: November 2013	Hypercholesterolemia, on background therapy with diet or ATV (10 mg or 80 mg) or EZE singly or in combination.	E-mAb versus Placebo	50.1% versus 6.8%	74.8% versus 74.2%

(3)	GAUSS-2 Completion date: November 2013	Hypercholesterolemia	E-mAb versus EZE	53–56% versus 37–39%	Muscle AE's; more frequent in EZE group (23%) versus E-mab group (12%)

(4)	RUTHERFORD-2 Primary completion date: November 2013	Familial Hypercholesterolemia	E-mAb versus placebo	60% in E-mAb group	Similar adverse events profile

(5)	LAPLACE-2 Completion date: Dec. 2013	Hypercholesterolemia	E-mAb versus EZE versus Placebo	−66% to 75% in E-mAb group	36% versus 40% versus 39%

(6)	FOURIER Completion date: 2017	Patient with h/o CVD on maximum tolerated statin therapy but LDL is more than 70 mg/dl	E-mAb versus placebo in statin treated patients	59% reduction of LDL in comparison to placebo	Primary end point. that is, Cv events was 9.8% versus 11.3%; Injection site reaction was more in E-mAb 2.1% versus 1.6%

(7)	OSLER I & OSLER II Expected completion date: June 2018 & August 2018, respectively	Patients who completed “parent trials” of evolocumab and eligible patients were randomly assigned in 2 : 1 ratio to receive either evolocumab plus standard therapy or standard therapy alone	E-mAb versus standard therapy	61% in E-mAb group.	69.2% versus 64.8%

**Table 3:** Studies on bococizumab.

S. number	Trial	Participants	Comparison	LDL reduction	Adverse events	Comments
(1)	Dose ranging trial (NCT01592240)	Those with LDL-C > 80 mg/dl on stable statin therapy	Bococizumab versus placebo	54.2% versus 2.8%	Similar adverse events profile	Despite dose reduction in many subjects, bococizumab significantly reduced LDL-C across all the doses

(2)	SPIRE-1 and SPIRE-2	Those with background lipid-lowering treatment and have an LDL-C of >/ = 70 mg/dl (SPIRE-1) or LDL-C >/ = 100 mg/dl (SPIRE-2)	Bococizumab versus placebo	This study has been terminated. Completion date: Jan. 2017		Bococizumab being a humanized monoclonal antibody, a strong immune response was seen against it which mitigate the LDL-C lowering effect. Anti-drug Ab was seen in 48% patients and neutralizing Ab developed in 29% patients
